# Giant primary omental cyst: an unusual cause of pseudoascites and hydrocele in a child

**DOI:** 10.1093/jscr/rjae774

**Published:** 2024-12-12

**Authors:** Hanan Youssif Mohamed, Habib Ullah Joya, Amani N Alansari

**Affiliations:** Pediatric Surgery Department, Benghazi Medical Center, Benghazi District 8606891, Sha'biyat Region, Libya; Department of Pediatric Surgery, Hamad Medical Cooperation, Doha 2001, Qatar; Department of Pediatric Surgery, Hamad Medical Cooperation, Doha 2001, Qatar

**Keywords:** omental cystic lymphangioma, inguinoscrotal swelling, communicating hydrocele

## Abstract

Abdominal lymphangiomas are benign congenital abnormal dilatation and proliferation of lymphatic spaces primarily seen in children. The wide spectrum of symptoms challenges preoperative diagnoses. We present a rare case of a 2-year-old boy presented to pediatrician with massive abdominal distention and left scrotal swelling since the age of 1½ years. Diagnosed and treated as ascites, the patient was referred to pediatric surgery for ascitic tap and hydrocele repair. An abdominal CT scan revealed a giant omental cyst. Laparotomy was performed to excise the cyst extending into left scrotum, and deep left inguinal ring was closed from within. Lymphangioma was histopathologically confirmed. To the best of our knowledge to date, only one case of an omental cystic lymphangioma presenting as bilateral hydroceles without abdominal symptoms has been reported. Nevertheless, this case is unique for the presentation of pseudoascites and a unilateral hydrocele.

## Introduction

Omental cysts are rare benign intra-abdominal lymphangiomas, formed due to malformations of the lymphatic system. Lymphangiomas are primarily (78%) seen in children <5 years old, with an incidence of 1: 250 000 [1]. These mainly (95%) occur in the head, neck or axilla, with a few (5%) found in the lungs, mediastinum, adrenal glands, kidney and bone. Even less common places include the mesentery, omentum, retroperitoneum, gastrointestinal tract, spleen, liver and pancreas [2].

Omental cysts can be an incidental finding or may present as abdominal pain or a mass. The mass may be huge, simulating ascites. Rarely present as acute abdomen due to peritonitis, hemoperitoneum or volvulus. This varied spectrum of symptoms challenges diagnoses [1].

Complete surgical excision remains the gold standard and the final diagnosis is histopathologically confirmed [3]. We present a rare case of a 2 years old boy, initially diagnosed and treated as ascites, but finally proved to have a giant omental cyst, presenting as pseudoascites and left sided hydrocele since the age of 6 months.

## Case presentation

**Figure 1:**
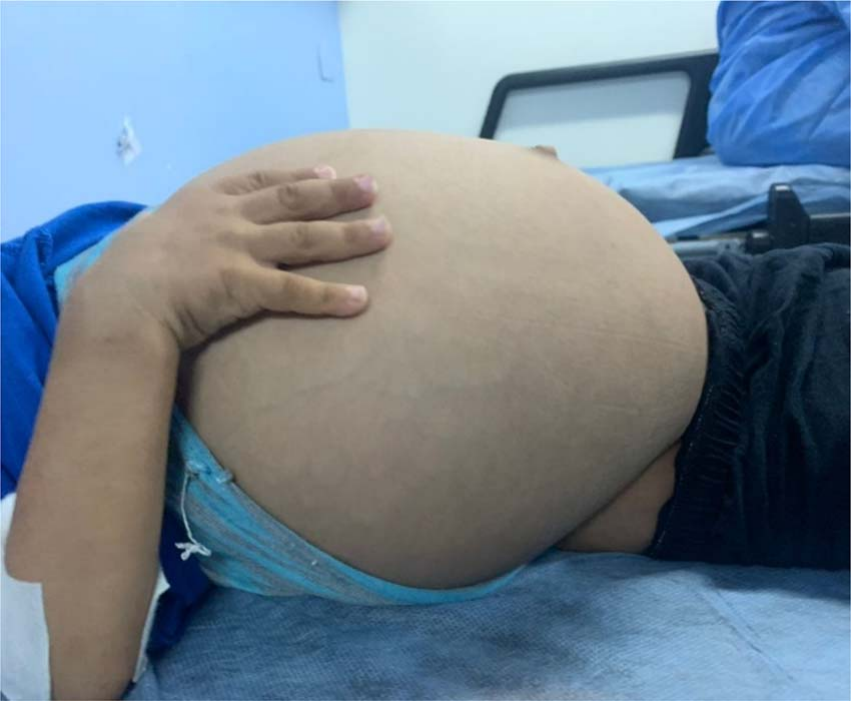
Massive abdominal distention mimicking pseudoascites.

**Figure 2:**
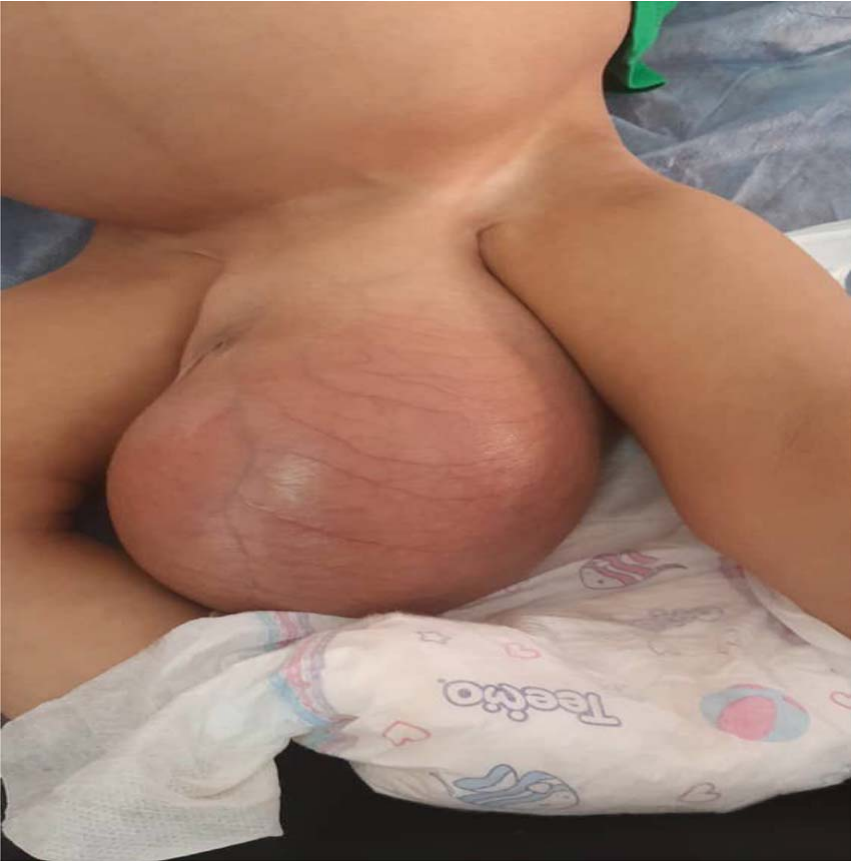
Hydrocele of left scrotum. Cyst extends through left inguinal canal. Completely buried penile shaft.

A 2 years old boy presented in pediatric emergency at his local hospital with painless abdominal distention and left scrotal swelling of gradual onset over the last 1½ years. The child was unable to walk. No history of infectious or other gastrointestinal symptoms. No history of trauma. His physical examination revealed a massively distended abdomen and visible veins without organomegaly (Fig. 1). Scrotal examination revealed gross left inguinoscrotal swelling, non-tender with normal overlying skin (Fig. 2). The swelling was partially reducible but would refill instantaneously on release of the pressure. Transillumination was positive. The left testis could not be felt. The rest of his examination was normal.

**Figure 3:**
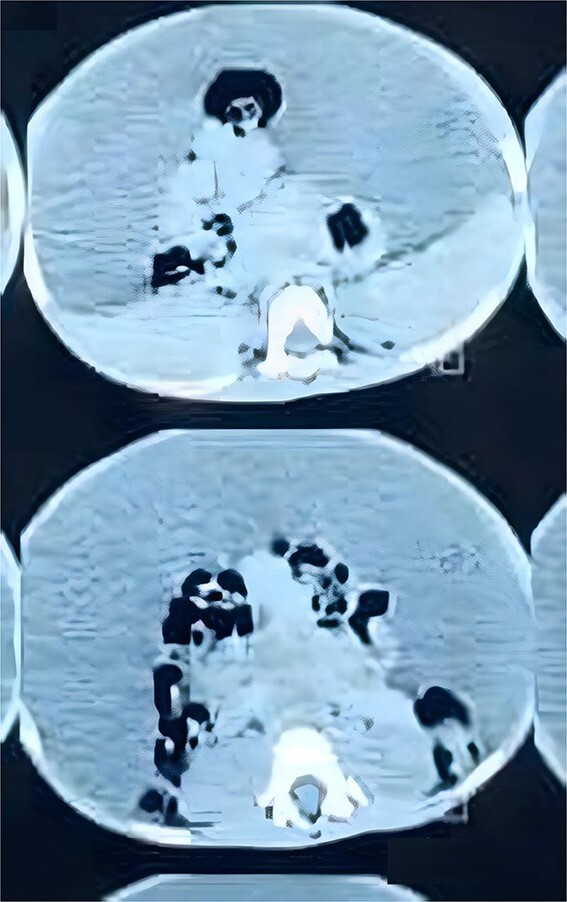
Axial view of CT abdomen and pelvis demonstrates the cystic lesion entrapping the bowel loops centrally

**Figure 4:**
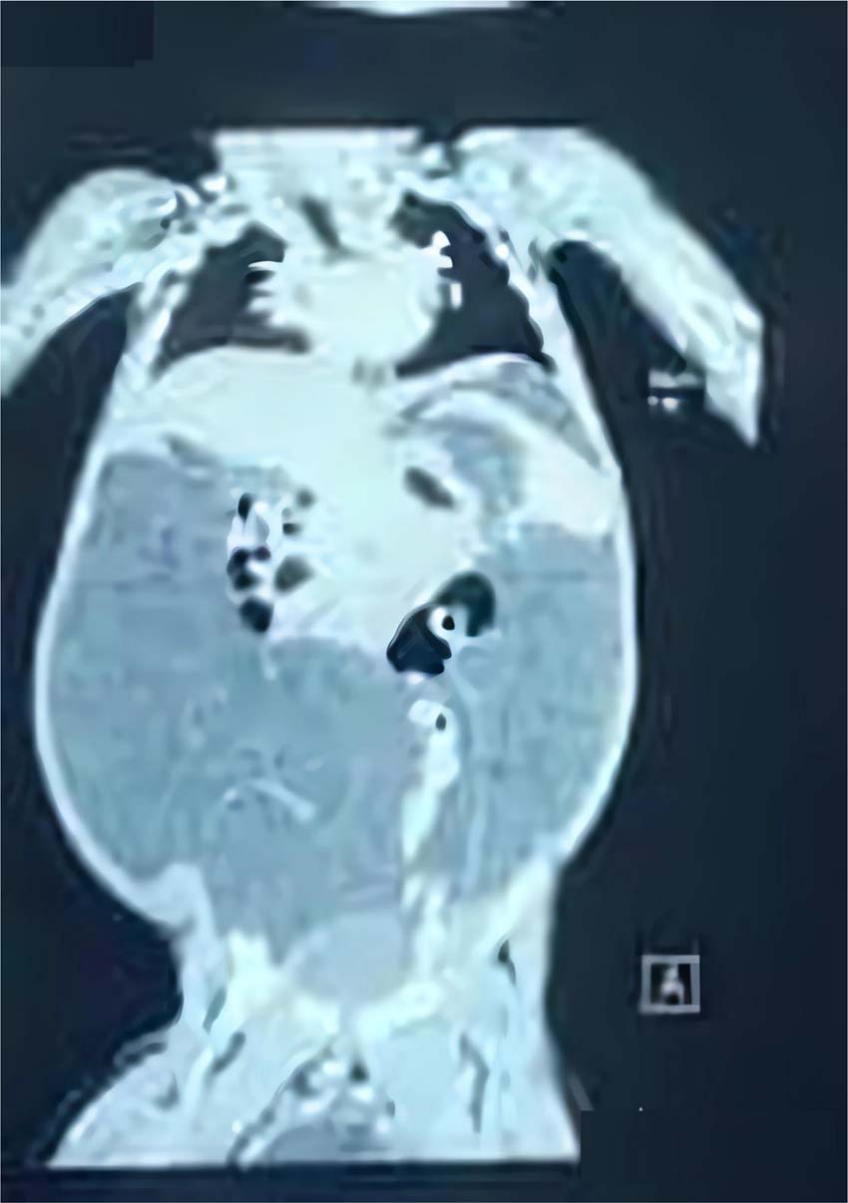
coronal view of CT abdomen and pelvic demonstrates the scrotal extent of abdominopelvic cystic lesion

His complete blood count, serum electrolytes, renal and liver function tests were within normal range. A large cystic multiloculated abdominoscrotal lesion was highlighted in the abdominal ultrasound. The abdomen and pelvis CT with contrast showed a huge hypodense cystic lesion around 25x30 cm with hyperdense multiple internal septations without solid component causing compression and posterior displacement of bowel loops (Fig. 3). It extended through the left inguinal canal into scrotum (Fig. 4). The patient was referred to the pediatric surgery department for reassessment. Subsequently, the decision was made to go for excision.

**Figure 5:**
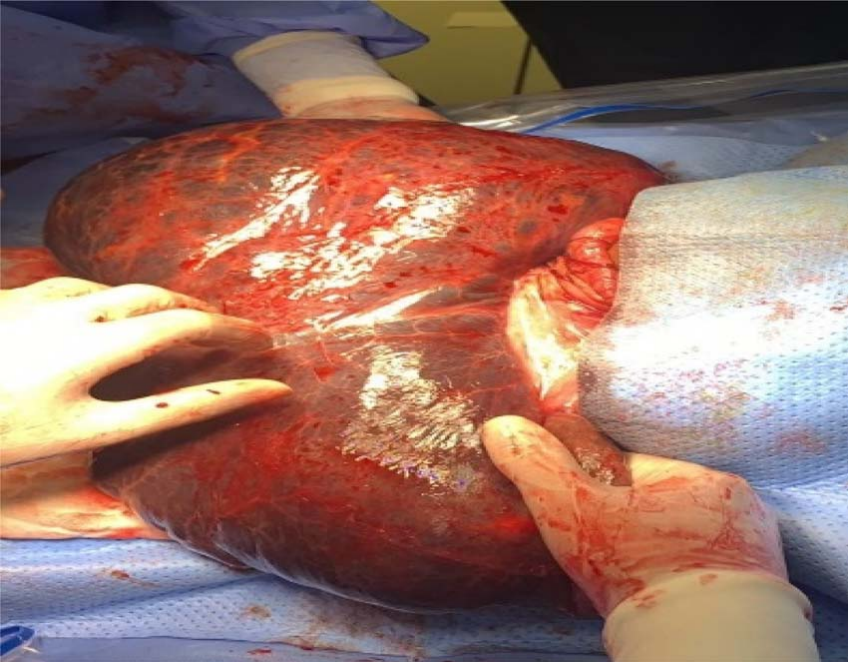
Omental cyst. Origin: greater omentum (arrow).

**Figure 6:**
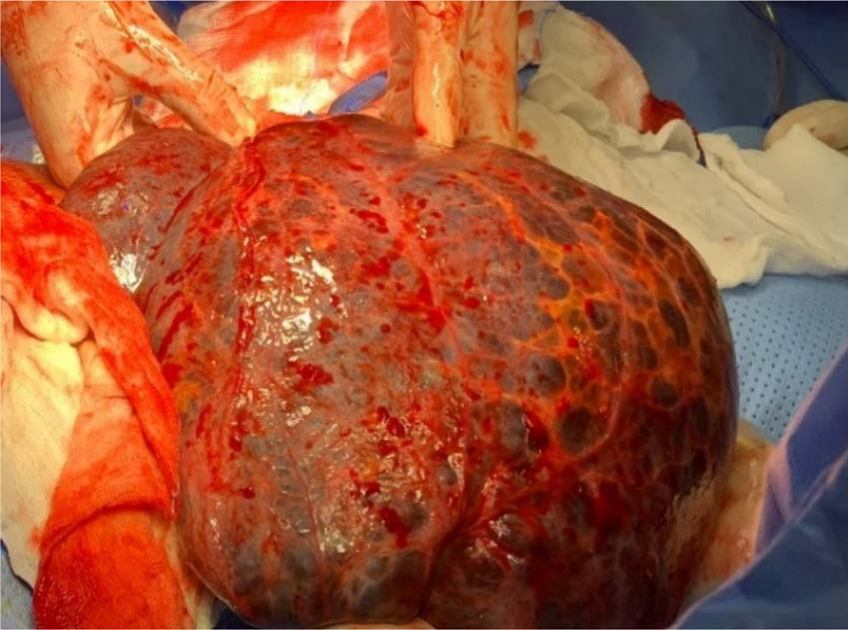
Intraoperative delivery of omental cyst for excision.

The patient underwent a median laparotomy. After opening the peritoneum, a large cyst arising from the greater omentum was noted (Fig. 5). It extended from subhepatic space to the left scrotum through the left inguinal canal. Approximately 25×30 cm large, it was multiloculated and contained straw- colored fluid (Fig. 6). Around 7 L of turbid yellowish fluid were evacuated to facilitate excision. It was neither adherent to the bowel nor other structures. The cyst was gently mobilized from the scrotal space and completely excised. The left hernia orifice was closed at the level of the deep inguinal ring, using purse string sutures.

Post-operatively, the patient was kept on antibiotics and IV fluid and allowed oral intake on the second postoperative day. The hospital course was uneventful. The patient was discharged on the 6th day. Histopathology confirmed the diagnosis of omental cystic lymphangioma. The patient received 3-monthly check-ups for an annum and annual thereafter. Currently, at the age of 5 years, the patient presents no signs of recurrence and is growing as a normal healthy child.

## Discussion

Intra-abdominal lymphangiomas are rare. Between the small bowel and retroperitoneal tissue, a lack of communication with the main lymphatic vessels forms a mass that may develop into mesenteric and omental lymphatic cysts [4]. Lesions located in the abdomen can grow without causing symptoms until later stages. [5].

Giant omental cysts represent a challenge for radiologists and surgeons. A high index of suspicion is required. The present case was initially diagnosed as ascites of unknown origin with left hydrocele. Referred to pediatric surgery for ascitic tap and hydrocele repair, the surgeon clinically suspected intraabdominal lymphangioma based on age and absence of any specific causes of ascites in this age. The CT abdomen was reviewed and in correlation with clinical suspicion, the diagnosis was made.

Despite the ability of ultrasound and computed tomography (CT) scans to assess the origin, size, and relationship of cysts with other structures, magnetic resonance imaging (MRI) is the most useful imaging modality. Intrinsic attenuation can vary from near water to fat attenuation, depending on the amount of chyle present [6]. US is suggestive of intra-abdominal cystic lymphangioma. However, in our case, the CT findings were sufficiently informative, and the findings were consistent with the CT report. Additionally, an MRI required patient transfer. Nevertheless, an MRI may have shortened the diagnosis and avoided multiple ascitic aspirations.

Complete excision is the treatment of choice to avoid risk of recurrence [7]. Some recent studies have demonstrated successful resection of abdominal lymphatic malformations using laparoscopy [8]. Considering the technical difficulties of such a huge mass and rate of recurrence, laparotomy was the most appropriate choice for this case.

Retroperitoneal lymphangioma is known to present as hydrocele, but the presentation of an omental cyst as abdominal distension and hydrocele is extremely rare. A case was initially diagnosed as retroperitoneal lymphangioma and intraoperatively found to originate from the greater omentum [9]. Another case reported an omental cyst with solely bilateral inguinoscrotal swelling without abdominal symptoms [10]. This first case from Libya is unique, as our patient presented an omental cyst with massive abdominal distension extending to the left scrotum, mimicking other causes of ascites and left hydrocele, the reason for its misdiagnosis as ascites of unknown origin. To the best of our knowledge, this is perhaps the first case of omental cystic lymphangioma presented with abdominal ascites and left hydrocele.

## Conclusion

Omental cystic lymphangioma is a rare cause of ascites in children. However, in the absence of hepatic, renal, or cardiac causes, there should be a high index of suspicion.

Abdomen US and CT scans are common diagnostic tools. If available, MRI presents more details regarding the cyst contents and is helpful in characterizing soft tissues. The preferred treatment is complete excision through either a laparotomy or a laparoscopic approach.

## Data Availability

Data will be made available on request.
